# *Citrus tristeza virus* p23: a unique protein mediating key virus–host interactions

**DOI:** 10.3389/fmicb.2013.00098

**Published:** 2013-05-03

**Authors:** Ricardo Flores, Susana Ruiz-Ruiz, Nuria Soler, Jesús Sánchez-Navarro, Carmen Fagoaga, Carmelo López, Luis Navarro, Pedro Moreno, Leandro Peña

**Affiliations:** ^1^Instituto de Biología Molecular y Celular de Plantas, Consejo Superior de investigaciones Científicas-Universidad Politécnica de ValenciaValencia, Spain; ^2^Centro de Protección Vegetal y Biotecnología, Instituto Valenciano de Investigaciones AgrariasMoncada, Valencia, Spain; ^3^Instituto de Conservación y Mejora de la Agrodiversidad Valenciana, Universidad Politécnica de ValenciaValencia, Spain

**Keywords:** closteroviruses, nucleolar proteins, RNA silencing suppressor, small interfering RNAs, zinc-finger domain

## Abstract

The large RNA genome of *Citrus tristeza virus* (CTV; ca. 20 kb) contains 12 open reading frames, with the 3′-terminal one corresponding to a protein of 209 amino acids (p23) that is expressed from an abundant subgenomic RNA. p23, an RNA-binding protein with a putative zinc-finger domain and some basic motifs, is unique to CTV because no homologs have been found in other closteroviruses, including the type species of the genus *Beet yellows virus* (despite both viruses having many homologous genes). Consequently, p23 might have evolved for the specific interaction of CTV with its citrus hosts. From a functional perspective p23 has been involved in many roles: (i) regulation of the asymmetrical accumulation of CTV RNA strands, (ii) induction of the seedling yellows syndrome in sour orange and grapefruit, (iii) intracellular suppression of RNA silencing, (iv) elicitation of CTV-like symptoms when expressed ectopically as a transgene in several *Citrus* spp., and (v) enhancement of systemic infection (and virus accumulation) in sour orange and CTV release from the phloem in p23-expressing transgenic sweet and sour orange. Moreover, transformation of Mexican lime with intron-hairpin constructs designed for the co-inactivation of p23 and the two other CTV silencing suppressors results in complete resistance against the homologous virus. From a cellular point of view, recent data indicate that p23 accumulates preferentially in the nucleolus, being the first closterovirus protein with such a subcellular localization, as well as in plasmodesmata. These major accumulation sites most likely determine some of the functional roles of p23.

## INTRODUCTION

**FIGURE 1:**
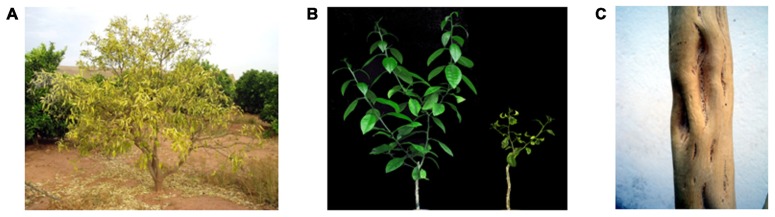
**Syndromes incited by CTV**. **(A)** Quick decline of sweet orange grafted on sour orange rootstock. **(B)** Seedling yellows expressed in Duncan grapefruit (right) compared with a non-infected control (left). **(C)** Stem pitting in sweet orange.

*Citrus tristeza virus* (CTV) – a member of the genus *Closterovirus*, family Closteroviridae – features singular biological and physical properties. In natural infections it is confined to the phloem of most species of the genera *Citrus* and *Fortunella* within the subfamily *Aurantioideae* (Bar-Joseph et al., 1989; Moreno et al., 2008), although when inoculated experimentally via *Agrobacterium tumefaciens* it may incite systemic infection and symptoms into the presumed non-host *Nicotiana benthamiana* (Ambrós et al., 2011). CTV may cause three different syndromes depending on the virus isolate, the citrus genotype, and the scion/rootstock combination. Tristeza, a bud-union problem causing phloem necrosis and decline of most citrus species propagated on sour orange (*Citrus aurantium* L.) rootstock, was the first syndrome observed in the 1930s’ decade, after this rootstock species became massively used worldwide to avoid root rot caused by oomycetes of the genus *Phytophthora*. In addition, some CTV isolates incite yellowing, stunting, and occasional growth arrest of sour orange, lemon (*C. limon* (L.) Burm. f.), and grapefruit (*C. paradisi* Macf.) seedlings [referred to as the seedling yellows (SY) syndrome], and/or stem pitting (SP; uneven radial growth with local depressions) on different citrus species irrespective of the rootstock used for their propagation, reducing the vigor, production, and fruit quality (**Figure 1**; Moreno et al., 2008).

**FIGURE 2:**
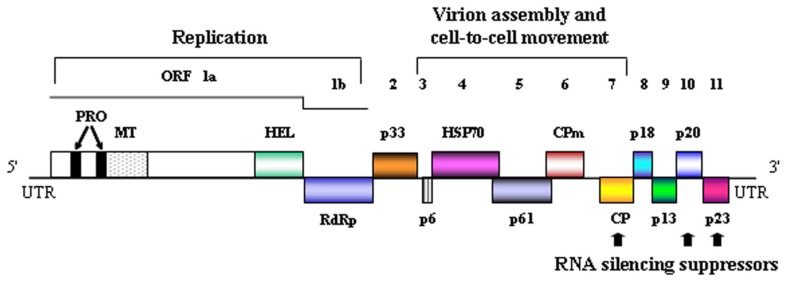
**Genomic organization of CTV**. Schematic representation of the genomic CTV RNA with boxes denoting open reading frames (ORFs) flanked by untranslated regions (UTRs). ORFs 1a and 1b contain several domains: PRO, papain-like protease; MT, methyltransferase; HEL, helicase; RdRp, RNA-dependent RNA polymerase. The functional role of some of the protein products is indicated. HSP70, CPm, and CP refer to a homolog of the plant heat shock protein 70 and to the minor and major coat proteins, respectively.

CTV is also peculiar from a physical standpoint because its monopartite, single-stranded RNA (ssRNA) (+) genome (19.3 kb), the largest reported for a plant virus, is organized in 12 open reading frames (ORFs) potentially encoding at least 17 proteins, confined between 5′ and 3′ untranslated regions (UTRs; **Figure 2**; Karasev et al., 1995; Mawassi et al., 1996; Vives et al., 1999; Yang et al., 1999). Remarkably, while the 3′ moieties of the genomic RNA (gRNA) from distinct CTV isolates display high sequence identity (89–97%), the corresponding 5′ moieties differ significantly (60–70%), with the difference increasing at the 5′UTR (Mawassi et al., 1996; López et al., 1998; Bar-Joseph and Dawson, 2008). The two 5′-proximal ORFs encoding components of the replicase complex are translated directly from the gRNA, with the remaining ORFs being expressed via ten 3′ co-terminal subgenomic RNAs (sgRNAs) differing in their accumulation and time course appearance during the infection process (Hilf et al., 1995; Navas-Castillo et al., 1997). Prominent among the resulting proteins, which mediate different aspects of the virus biology, is p23, encoded by the 3′-terminal ORF (**Figure 2**). The absence of homologs in other closteroviruses, including the type species of the genus *Beet yellows virus* (BYV) with which CTV has many homologous genes (Dolja et al., 2006), suggests that p23 might have evolved for regulating specific interactions of CTV with its citrus hosts.

In the following sections, we will review the structural and biochemical properties of p23, the different known roles in CTV biology played by this multifunctional protein, and its major subcellular accumulation sites. Some of these roles are most likely interconnected with one another and dependent on the intracellular locations of p23.

## STRUCTURAL AND BIOCHEMICAL PROPERTIES

**FIGURE 3:**
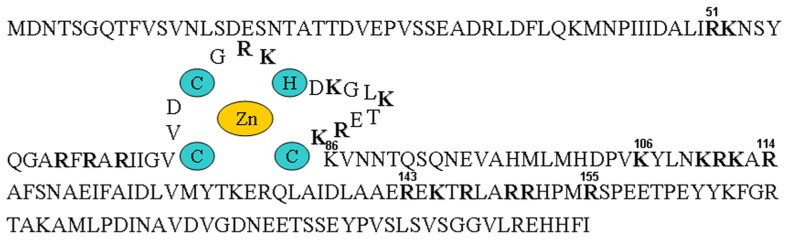
**Biochemical and structural properties of p23**. Outline of the amino acid sequence of p23 from strain T36, with the putative zinc-finger domain and the coordinating histidine and cysteines highlighted with colored background, and the arginines and lysines forming part of motifs rich in basic amino acids denoted with bold fonts.

Analysis of many different CTV isolates has revealed a constant size for p23 of 209 amino acids, resulting in a molecular mass of approximately 23 kDa. Although no significant similarity was observed between p23 and other amino acid sequences deposited in databases (Pappu et al., 1994), a basic motif and some conserved cysteines, also found in proteins with RNA-binding properties encoded at the 3′-proximal region of the gRNA of some filamentous viruses (see below), suggested that p23 might also have RNA-binding properties and be engaged in regulating viral gene expression (Dolja et al., 1994). A closer inspection revealed the conserved motif CVDCGRKHDKALKTERKC between amino acids 68 and 85, with the underlined cysteines and histidine – and their relative positions – making highly feasible the coordination of a Zn ion by adopting a tetrahedral zinc-finger domain (**Figure 3**; López et al., 1998). Subsequent examination has confirmed that the three cysteines and the histidine that potentially coordinate the Zn ion are strictly conserved, as well as most of the flanking basic amino acids between positions 50 and 86, and some additional basic motifs (Sambade et al., 2003). Furthermore, while the N-terminal region of p23 has a net positive charge (the p*I* of the segment delimited by amino acids 29–155 is close to 11), the C-terminal region has a net negative charge (the p*I* of the segment corresponding to the last 54 amino acids is 4.35). This asymmetrical charge distribution, and the putative zinc-finger domain, have been observed in the transactivating domains of some transcription factors (López et al., 1998).

The predicted RNA-binding properties of p23 were confirmed following its expression in *Escherichia coli*, fused to the maltose-binding protein, and purification by affinity chromatography (López et al., 2000). Gel retardation and UV crosslinking experiments showed that p23 binds ssRNA cooperatively and in a non-sequence-specific manner. Even if formation of the p23–RNA complex depends on the conformational state of p23 and on the presence of at least one basic motif, this complex is stable at high salt concentrations, suggesting the involvement of interactions other than those between the basic motifs of p23 and the negatively charged RNA. Competition assays showed a clear higher affinity of p23 for ssRNA and double-stranded RNA (dsRNA) than for their DNA counterparts, although the affinity for both RNA types was similar. Mapping studies with deletion mutants demarcated the RNA-binding domain of p23 to the segment between positions 50 and 86, containing the putative zinc-finger domain and motifs rich in basic amino acids. Additional p23-derivatives lacking the conserved amino acids predicted to coordinate the zinc ion displayed RNA-binding activity, although with an apparent dissociation constant higher than the wild-type protein, suggesting that those amino acids might provide increased binding stability or specificity *in vivo* (López et al., 2000).

## REGULATION OF THE ASYMMETRICAL ACCUMULATION OF CTV RNA STRANDS

Analysis of the accumulation kinetics of CTV RNAs in *N. benthamiana* protoplasts revealed that the gRNA increased to a maximum at 3–5 days after transfection, with the full-length minus RNA increasing with similar kinetics but at approximately one-tenth the level of the plus strand RNA. Accumulation of most sgRNAs paralleled that of the gRNA, but their levels differed considerably. Interestingly, the sgRNA corresponding to p23 reached the highest level (similar to that of the gRNA), and increased earlier than other sgRNAs (Navas-Castillo et al., 1997), an observation consistent with a regulatory role for p23.

The finding that CTV infection results in the production of more plus than minus strands (of both genomic and subgenomic length) is typical of ssRNA viruses. However, this asymmetrical proportion was perturbed in a CTV mutant replicon with all of the 3′ genes deleted, which replicated efficiently in *N. benthamiana* protoplasts but resulted in an almost equal ratio of plus to minus strands (Satyanarayana et al., 2002). Because a frameshift mutation in one of the first codons of the 3′-proximal ORF caused essentially the same effect, p23 itself, an not the RNA fragment coding for it, appeared to control the asymmetrical balance of CTV strands. Data obtained with additional in-frame deletion mutants of this ORF indicated that both terminal regions (delimited by amino acids 5–45 and 181–209) were dispensable, while the central region (from amino acid 46 to 180, containing the zinc-finger domain and RNA-binding motifs; **Figure 3**), was required for asymmetrical RNA accumulation. Further alanine substitution mutants mapped the conserved cysteines in the zinc-finger domain as critical for this regulatory activity of p23. Moreover, whereas non-functional p23 essentially resulted in a significant overaccumulation of minus strands, functional p23 induced a decrease in the accumulation of the minus-stranded sgRNA corresponding to the major coat protein (p25) but not in the accumulation of this protein (which was substantially increased), suggesting that the availability of the corresponding plus-stranded sgRNA as a messenger is blocked by the excess of its minus-stranded counterpart. In summary, by downregulating minus-stranded RNA accumulation (and increasing indirectly expression of the 3′ genes), p23 would control the unbalanced accumulation of CTV RNAs (Satyanarayana et al., 2002).

## ASSOCIATION WITH THE SEEDLING YELLOWS SYNDROME IN SOUR ORANGE AND GRAPEFRUIT

As indicated above, some CTV isolates incite the SY syndrome in sour orange, lemon, and grapefruit (**Figure 1**), which occasionally may be transitory with the plants resuming normal growth after some time. Although economically not relevant, this syndrome can be studied under greenhouse conditions more easily than decline and SP. The construction of an infectious CTV-cDNA clone (Satyanarayana et al., 1999, 2001) opened the possibility of dissecting the pathogenicity determinant of SY. Instrumental for this purpose were an isolate of the decline strain T36, which additionally induces SY, and an isolate of the mild strain T30, which does not. By replacing in an infectious full-length cDNA of T36 different regions of its 3′-moiety with the corresponding T30 sequences, eleven T36/T30 recombinant clones were obtained able to replicate in protoplasts of *N. benthamiana.* Five of these hybrids produced virions that were inoculated mechanically to alemow (*C. macrophylla* Wester), a sensitive CTV host, resulting in systemic infections. When tissues from these infected plants were graft-inoculated into sour orange and grapefruit seedlings, three of the T30/T36 recombinants incited SY symptoms identical to those of T36, whereas the other two – with T30 substitutions in the p23-3′UTR – did not. Moreover, the presence of a non-SY p23-3′UTR recombinant in sour orange seedlings resulted in their protection against challenge inoculation with the parental T36 virus, thus showing the potential for cross-protection of the engineered CTV constructs (Albiach-Martí et al., 2010). Because the 3′UTR is highly conserved among all CTV genotypes (Mawassi et al., 1996; López et al., 1998), p23 is a plausible candidate for the pathogenicity determinant of SY (and perhaps for the decline syndrome). Moreover, p23 is an intracellular suppressor of RNA silencing and, as such, with potential for disrupting developmental pathways mediated by small RNAs (see next section).

## INTRACELLULAR SUPPRESSION OF RNA SILENCING

RNA gel-blot analyses have revealed that, in addition to the gRNA and sgRNAs, CTV infection of Mexican lime [*C. aurantifolia* (Christm.) Swing.], another particularly sensitive host, results in the high accumulation of virus-derived small RNAs (vsRNAs) (Fagoaga et al., 2006; see also below). The latter belong to a broader class of small RNAs (sRNAs) – including micro RNAs (miRNAs) of 21 and 22 nt and small interfering RNAs (siRNAs) of 21, 22, and 24 nt – that are the hallmark of RNA silencing, a regulatory mechanism for modulating host gene expression and protecting plants and most other eukaryotes against invading nucleic acids, both foreign (viruses and transgenes) and endogenous (transposons). RNA silencing is triggered by dsRNAs and snap-folded ssRNAs that are processed into sRNAs by particular RNase III isozymes (Dicer or Dicer-like, DCL, in plants; Carthew and Sontheimer, 2009; Chen, 2009). The sRNAs subsequently load and guide specific Argonaute (AGO) proteins, at the core of the RNA inducing silencing complex, for inactivating their cognate DNAs and RNAs at the transcriptional and post-transcriptional level (Mallory and Vaucheret, 2010). To cope with this mechanism, viruses have evolved to encode in their genomes RNA silencing suppressors (RSS) that perturb one or more steps thereof (Csorba et al., 2009; Ding, 2010). Since the defensive branch of RNA silencing overlaps to some extent with another branch regulating plant homeostasis via miRNAs and siRNAs, the developmental alterations caused by viruses are deemed lateral effects of their RSS acting on both branches (Kasschau et al., 2003; Jay et al., 2011), although not all viral symptoms must inevitably have this origin (Díaz-Pendón and Ding, 2008).

To identify and characterize the RSS in CTV, nine of the proteins encoded in the 3′ moiety of the gRNA were co-agroexpressed, together with the green fluorescent protein (GFP) in a transgenic line of *N. benthamiana* (16c) expressing GFP constitutively (Lu et al., 2004). Analysis of GFP in the infiltrated leaves by fluorescence and RNA gel-blot hybridization revealed strong and moderate RSS activity for p23 and p20, respectively, as well as occasional partial suppression of systemic silencing in plants co-infiltrated with the p25 construct (despite this protein not functioning as a RSS in the infiltrated leaves). Further examination was performed in an independent silencing system based on the GUS (beta-glucuronidase) transgene in a tobacco line (6b5), wherein silencing of the transgene takes place autonomously and persistently in each generation. Following their introduction by genetic crosses into this line, the effect of p23, p20, and p25 on the intracellular and intercellular silencing of the GUS transgene was analyzed in the F1 progeny: abundant accumulation of the GUS-mRNA was detected in p20 × 6b5 and, particularly, in p23 × 6b5 plants, but not in their p25 × 6b5 counterparts, thus indicating that the two former, but not the latter, function as intracellular RSS. Subsequent grafting experiments in which the tobacco line T19 expressing GUS was propagated onto control 6b5 and p23 × 6b5 rootstocks showed that the corresponding scions became silenced (as illustrated by the accumulation of GUS-specific siRNAs and the decrease of GUS-mRNA), hence indicating that p23 did not interfere with the production or export of the GUS-specific silencing signal from the 6b5 rootstock locus into the T19 scions. Conversely, accumulation of GUS-mRNA and lack of GUS-siRNAs were observed in T19 scions grafted onto either P20 × 6b5 or CP × 6b5 rootstocks. Altogether these findings showed that p23 is a suppressor of just intracellular silencing, p25 of just intercellular silencing, and p20 of both types of silencing, thus unveiling that CTV has evolved an elaborated viral counter-defense directed against several steps of the silencing antiviral route, a strategy most likely needed for protecting such a large gRNA (Lu et al., 2004). The RSS activity of p23 has been examined in more detail recently (see below).

To complement these studies and getting a deeper insight into the CTV-induced RNA silencing response, as well as on the counter defensive reaction mediated by its three RSS, the sRNA patterns were analyzed by gel-blot hybridization, deep sequencing (Solexa-Illumina), and bioinformatic approaches in young bark of three natural hosts infected by a severe CTV isolate, as well as in their corresponding mock-inoculated controls. The data obtained (Ruiz-Ruiz et al., 2011) show that CTV sRNAs: (i) are very abundant (more than 50% of the total sRNAs, indicative of a strong antiviral response) in Mexican lime and sweet orange [*C. sinensis* (L.) Osb.; in which the virus titer is relatively high], but only amount to 3.5% in sour orange (with a considerably lower CTV titer), (ii) have a predominant size of 22- 21-nt, with an uneven balance in their 5′ nucleotide and a moderate overaccumulation of those of (+) polarity, and (iii) result from all the CTV genome, permitting its entire assembly from viral sRNA contigs, but display an asymmetrical profile characterized by a major hotspot corresponding to the 3′-terminal 2500 nt. These observations suggest that the genesis of the 22- and 21-nt CTV sRNAs is most likely catalyzed by the citrus homologs of DCL 2 and 4, respectively, and that these two ribonuclease isoenzymes operate on the gRNA as well as on the 3′ co-terminal sgRNAs, and, particularly, on their double-stranded forms. In the three mock-inoculated controls, the sRNA pattern was very similar and dominated by the 24-nt sRNAs. This pattern remained essentially unaffected by CTV infection in sour orange, while a clear reduction of the 24-nt sRNAs was detected in Mexican lime and sweet orange. In addition, CTV influences the accumulation of some miRNAs. For instance, miRNA 168 is upregulated by CTV in the three hosts (Ruiz-Ruiz et al., 2011). This miRNA targets the mRNA coding for AGO1, which mediates miRNA-directed (Baumberger and Baulcombe, 2005) and vsRNA-directed silencing (Morel et al., 2002). While AGO1 accumulation results from the host defensive response, miR168 induction – detected in distinct plant–virus combinations – appears a counter-defense reaction of the virus (Varallyay et al., 2010).

## INDUCTION OF CTV-LIKE SYMPTOMS WHEN EXPRESSED TRANSGENICALLY IN SEVERAL CITRUS SPECIES

**FIGURE 4:**
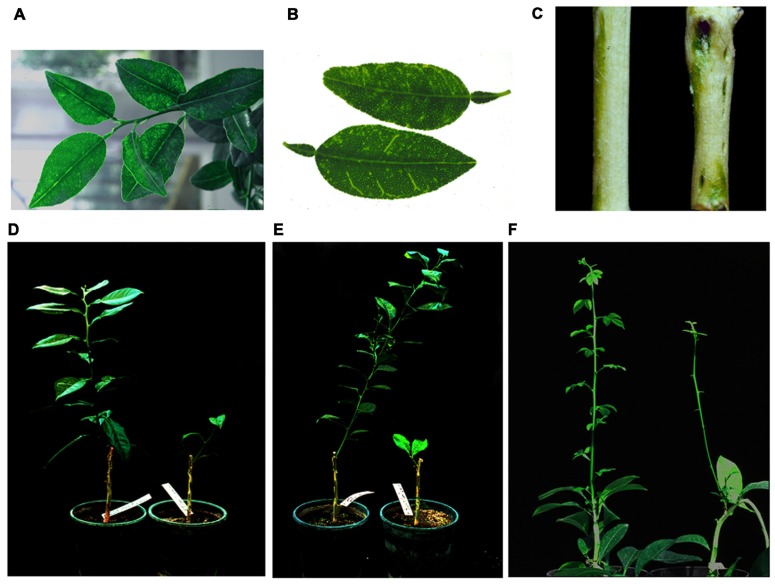
**Phenotypic aberrations resulting from the transgenic expression of p23 in citrus plants**. **(A)** Vein clearing and chlorotic pinpoints in Mexican lime expressing p23. **(B)** Leaves from a non-transgenic Mexican lime inoculated with a severe CTV strain (top) and from a non-inoculated transgenic Mexican lime expressing p23 (bottom). **(C)** Stem pitting and normal appearance in Mexican lime transformed with a vector expressing p23 (right) and with the empty vector control (left), respectively. **(D)** Leaf distortion and stunting of a sour orange plant expressing transgenically wild-type p23 (right) compared with the normal appearance of a plant expressing a truncated version of this protein (left). **(E)** The same as in **(D)** but referred to sweet orange. **(F)** Developmental aberrations, including leaf abscission and stunting, of a trifoliate orange plant expressing transgenically wild-type p23 (right) compared with the normal appearance of a plant expressing a truncated version of this protein (left).

Before their role as RSS was elucidated, many viral proteins of this class were identified as pathogenicity determinants, because in addition to blocking the host defensive response RSS often interfere with host developmental pathways mediated by endogenous sRNAs (see above). This was also the case with p23, the deduced amino acid sequence of which correlated in phylogenetic reconstructions with mild and severe strains (Pappu et al., 1997; Sambade et al., 2003). Interestingly, ectopic expression in Mexican lime of p23 (strain T36) under the control of the 35S promoter from *Cauliflower mosaic virus* (CaMV) resulted in phenotypic aberrations – including intense vein clearing, general epinasty and chlorotic pinpoints in leaves, pitting and necrosis in the stems, and collapse – even more pronounced than those caused by CTV in non-transgenic plants (vein clearing, epinasty of emerging leaves that later evolves in leaf cupping, and SP), whereas Mexican limes expressing transgenically a truncated version of p23 were normal (**Figure 4**). Therefore, induction of CTV-like symptoms was associated with the expression of p23, and its accumulation level paralleled symptom intensity (Ghorbel et al., 2001). An extension of this study to other citrus hosts revealed that transformation with p23 of the CTV-susceptible sour and sweet orange, and of the CTV-resistant trifoliate orange [*Poncirus trifoliata* (L.) Raf.], also incited CTV-like leaf symptoms (vein clearing, epinasty, and stunting in sour and sweet orange, and chlorosis, leaf abscission, stem necrosis, stunting, and apical necrosis in trifoliate orange), which again were not expressed in the controls transformed with a truncated p23 version (**Figure 4**). However, in contrast with the situation observed in Mexican lime, p23 was nearly undetectable in transgenic sour, sweet, and trifoliate orange, although symptom intensity correlated with levels of the p23 transcript. Because p23 also accumulates poorly in non-transgenic sweet and sour orange inoculated with CTV in comparison with Mexican lime, these results suggest that even minimal levels of p23 may induce toxic effects in the two former species. On the other hand, transgenic expression of p23 in *N. benthamiana* and *N. tabacum* resulted in the accumulation of p23 without visible phenotypic aberrations, indicating that p23 interference with plant development is citrus-specific (Fagoaga et al., 2005).

Also in this context, examination of the phenotypic aberrations resulting from the transgenic expression of p23 under the control of a phloem-specific promoter should better mimic the symptoms incited by CTV, which is a phloem-confined virus. Indeed, it will be interesting to see whether restricting the expression of p23-derived transgenes to phloem-associated cells of Mexican lime by using a promoter from *Commelina yellow mottle virus* (Medberry et al., 1992) results in phenotypes more closely resembling symptoms induced by virus infection, because the other abnormalities observed previously may well be pleiotropic effects derived from p23 accumulation in non-phloem cells. There are experiments in progress aimed at examining this and another two related questions: (i) whether some of the CTV-like symptoms induced by the phloem-specific expression of p23 from strain T36 are not induced by p23 from the milder strain T317, in contrast with the similar effects observed when both protein variants are expressed constitutively (Fagoaga et al., 2005) and, (ii) whether expression in phloem tissues of the p23 fragment comprising the zinc-finger domain and flanking basic motifs is sufficient to induce CTV-like symptoms, corroborating that the N-terminal region (delimited by amino acids 1 and 157; **Figure 3**) determines, at least in part, CTV pathogenesis in Mexican lime.

## TRANSGENIC EXPRESSION OF p23 INCREASES CTV ACCUMULATION IN SOUR ORANGE AND PROMOTES VIRUS RELEASE FROM THE PHLOEM IN SWEET AND SOUR ORANGE

Analysis of CTV distribution in two citrus species after inoculation with the virions resulting from transfecting protoplasts of *N. benthamiana* with the transcript from an infectious CTV-cDNA clone (strain T36) expressing the GFP as an extra ORF (Satyanarayana et al., 2001; Folimonov et al., 2007) revealed that the infection foci comprise clusters of multiple cells in the highly susceptible host alemow, in contrast to the single-cell foci usually observed in the less-susceptible host sour orange. These results suggest lack of intercellular movement in sour orange, in which CTV infection would represent an extreme situation, with the virus apparently relying only on the long-distance movement (Folimonova et al., 2008).

To get a deeper insight into this question, transgenic plants of sweet orange (a highly susceptible host) and sour orange expressing ectopically p23 (Fagoaga et al., 2005) were graft-inoculated with the mild CTV isolate T385 or with the GFP-tagged CTV clonal strain derived from isolate T36. While CTV accumulation in p23-expressing and control (transformed with an empty vector) sweet orange was similar, the viral load was several times higher in transgenic sour orange expressing p23 than in the corresponding control plants. Moreover, in contrast with the few single-cell infection foci detected in the phloem of CTV-infected sour orange controls, in p23-expressing sour orange the number of foci was higher and included generally two to six cells, thus indicating intercellular movement of the virus. On the other hand, CTV infection in p23-expressing plants was not restricted exclusively to phloem tissues, since GFP-derived fluorescence was observed in some mesophyll protoplasts and cells from infected sour and sweet orange expressing p23, but not in similar protoplasts and cells from infected controls (Fagoaga et al., 2011).

Altogether these results show that, when expressed ectopically, p23 facilitates CTV phloem escaping and additionally enhances systemic infection of the less-susceptible sour orange host. Moreover, the distinct reaction observed in sweet and sour orange implies a multifaceted interaction between p23 and other virus- and host-encoded proteins for traversing diverse cell boundaries. In this context, the possibility that CTV exit from the vascular system in sweet and sour orange could be mediated by a p23 function unrelated to silencing suppression should be entertained. The finding that p23 accumulates preferentially in plasmodesmata, in a addition to the nucleolus (see below), is consistent with the involvement of this protein in CTV cell-to-cell and long-distance movement (Fagoaga et al., 2011).

## TRANSGENIC PROTECTION MEDIATED BY RNA SILENCING

The initial aim of overexpressing p23 in transgenic citrus (see above) was to test whether, under such conditions, this regulatory protein could interfere with CTV replication and provide resistance. Although most transgenic lines showed phenotypic aberrations similar to CTV symptoms (see above), certain lines of Mexican lime transformed with gene *p23* appeared normal and manifested the typical features of RNA silencing: multiple copies and methylation of the transgene, presence of *p23*-specific siRNAs, and low accumulation of the corresponding mRNA. CTV inoculation, by grafting or aphids, of propagations from these latter lines resulted in three responses: some were immune (they neither expressed symptoms nor accumulated the virus), others were partially resistant (with delayed and attenuated symptoms compared to controls transformed with the empty vector), and a third group was susceptible (with symptoms and virus titer similar to the controls). This erratic response of clonal propagations denoted that RNA-mediated resistance is also influenced by factors other than the transgenic background, like the developmental stage or growing conditions of each plant (Fagoaga et al., 2006).

It has been known for some time that intron-hairpin constructs containing virus sequences induce a strong antiviral reaction; this is because the transcribed dsRNA triggers RNA silencing that ultimately results in transgene-derived siRNAs and inactivation of the cognate viral ssRNA (Smith et al., 2000). Based on this finding, a 549-nt CTV sequence comprising part of gene *p23* and the 3′UTR of the gRNA, a segment highly conserved (>90% identity) in different virus isolates, was used to transform Mexican lime in intron-hairpin, sense, and antisense formats. When graft-inoculated with CTV, all propagations from sense, antisense, and empty-vector transgenic lines were virus-susceptible, except one (out of seven) from a sense-line showing transgene-derived siRNAs, low accumulation of the transgene-derived transcript, and an intricate transgene integration profile. In contrast, 9 of 30 intron-hairpin lines were partly resistant to CTV: 9–56% of their propagations (depending on the line) remained uninfected, with the others being susceptible. Focusing on intron-hairpin lines with a single-transgene integration (to facilitate comparison between lines), the low accumulation of the transgene-derived transcript was a better predictor of CTV resistance than the high accumulation of the transgene-derived siRNAs, possibly because only a part of the latter are competent for RNA silencing (López et al., 2010).

These and results from other groups (Batuman et al., 2006; Roy et al., 2006; Febres et al., 2008) illustrate that developing RNA silencing-based resistance against CTV has remained elusive. To move forward, Mexican lime was transformed with an intron-hairpin construct containing full-length, untranslatable versions of genes *p25*, *p20*, and *p23* (clonal strain T36) to silence concurrently the expression of the three RSS of CTV in infected cells. Graft-inoculation with an isolate of the same viral strain, in the scion or in the rootstock, revealed that three transgenic lines were completely resistant: all their propagations remained asymptomatic and virus-free, with the accumulation of transgene-derived siRNAs being necessary but insufficient for CTV resistance. However, resistance was only partial following inoculation with an isolate of a severe SP strain (T318A), with nucleotide identities with T36 of 91–92% for the three genes, thus showing the involvement of a sequence-dependent mechanism. Apart from representing a step ahead in the quest for developing full transgenic resistance to CTV, these results show that the simultaneous inactivation of the three viral RSS is crucial for this aim, although the participation of other concomitant RNA silencing mechanisms cannot be dismissed (Soler et al., 2012).

## NUCLEOLAR LOCALIZATION

**FIGURE 5:**
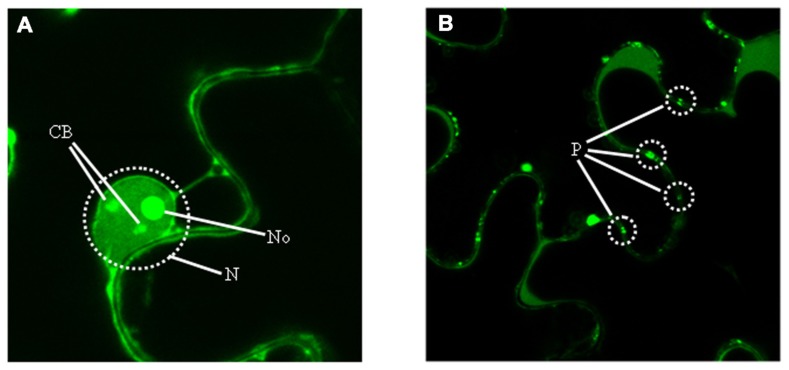
**Predominant subcellular accumulation of p23**. Examination with confocal laser-scanning microscopy of leaves from *Nicotiana benthamiana* agroinfiltrated with a construct expressing the recombinant protein p23–GFP, which accumulates predominantly in **(A)** two nuclear (N) compartments, the nucleolus (No) and Cajal bodies (CB), and in **(B)** plasmodesmata (P).

Because determining the subcellular localization site of a protein is central for understanding its biological role, the fusion p23–GFP was agroexpressed in leaves of *N. benthamiana*. Analysis of the infiltrated halos by confocal laser-scanning microscopy revealed the preferential accumulation of this recombinant protein in the nucleolus and in nucleolar bodies resembling Cajal bodies, as well as in punctuated structures at the cell wall similar to plasmodesmata (**Figure 5**). These results, confirmed by coexpression experiments with proteins marking specifically the nucleolus (fibrillarin) and plasmodesmata (the movement protein of an ilarvirus), strongly suggested the presence in p23 of a nucleolar localization signal (NoLS) and of a plasmodesmatal localization signal (PLS). Because NoLS (as well as nuclear localization signals) are formed by short motifs rich in basic amino acids, the possibility that motifs of this class identified previously in p23 might form part of its NoLS was examined. Assay of seven truncated versions of p23 fused to GFP showed that regions 50–86 and 100–157 (excluding fragment 106–114), both with basic motifs and the first with a predicted zinc-finger domain (**Figure 3**), contain what appears a bipartite NoLS. Additional data obtained with 10 alanine substitution mutants confirmed and delimited this signal to three cysteines of the zinc-finger domain and to some basic amino acids. It is also worth noting that, even if a fine dissection of the PLS was not carried out (in part because the molecular features of these signals are not well established), all deletion mutants (except one lacking the C-terminal fragment delimited by amino acids 158 and 209) lost their PLS (Ruiz-Ruiz et al., 2013).

As already indicated, p23 behaves as an RSS when co-agroexpressed with GFP – both under the control of the 35S promoter – in the transgenic line of *N. benthamiana* 16c expressing GFP also constitutively (Lu et al., 2004). However, while in leaves co-infiltrated with plasmids 35S-p23 and 35S-GFP the fluorescence remained intense 6–7 days later, it became almost undetectable in leaves infiltrated with just plasmid 35S-GFP, or co-infiltrated with either the empty plasmid or with any of the 17 plasmids expressing the individual p23 deletion and substitution mutants (with the exception of that affecting the histidine of the predicted zinc-finger domain). In accordance with these observations, gel-blot hybridizations with a GFP-specific riboprobe and RNA preparations from leaves emitting strong fluorescence revealed high and low accumulation of GFP-mRNA and GFP-siRNAs, respectively, whereas the reverse situation occurred using RNA preparations from leaves with undetectable fluorescence. Collectively these results showed that the RSS activity of p23 involves most regions of this protein (Ruiz-Ruiz et al., 2013).

Although p23 does not incite phenotypic aberrations when expressed transgenically in *N. benthamiana* (see above), the protein was additionally expressed as a sgRNA of *Potato virus X* (PVX) because different RSS act as pathogenicity determinants in this experimental context (Voinnet et al., 1999). Indeed, p23 induced a necrotic reaction in *N. benthamiana*, with this ability being preserved only in the deletion mutant lacking the C-terminal fragment delimited by amino acids 158 and 209 and in the substitution mutant affecting the histidine of the predicted zinc-finger domain. Therefore, this domain and its flanking basic motifs form part of the pathogenicity determinant. Ectopic expression of p23 and three deletion mutants thereof in transgenic Mexican lime delimited a similar determinant for symptom expression, suggesting that similar regions of p23 are associated with pathogenesis in *N. benthamiana* and citrus (Ruiz-Ruiz et al., 2013).

## CONCLUDING THOUGHTS AND PERSPECTIVES

Despite not being part of the virion, p23 has been more deeply analyzed than most other CTV-encoded proteins, with these studies unveiling a plethora of effects on the accumulation of CTV RNA strands, RNA silencing suppression, pathogenesis, and virus movement. The preferential location of p23 in the nucleolus (and Cajal bodies), as well as in plasmodesmata, is most likely instrumental for these effects. Considerable progress has been also achieved in dissecting the structural motifs of p23 associated with its functions. However, many facets of this singular protein await a deeper analysis, including its potential interactions with other proteins from the virus (like those catalyzing replication or mediating movement) and the host (including different AGOs involved in silencing), as well as with nucleic acids also of viral and host origin (for instance, the sRNAs that mediate silencing). These interactions should be ultimately mapped to specific p23 motifs/domains, as recently reported for other multifunctional virus proteins (Duan et al., 2012).

As noted above, p23 is unique to CTV within closteroviruses. However, the gRNAs of some filamentous viruses – from genera *Vitivirus*, *Carlavirus*, and *Benyvirus* – encode in their 3′-proximal region proteins containing basic amino acid motifs and a putative zinc-finger domain (Chiba et al., 2006, 2013). Some of these proteins, like p23, accumulate in the nucleolus (or in the nucleus), and display RNA-binding activity, suppression of RNA silencing, and induction of necrosis in *N. benthamiana* when launched from PVX (Lukhovitskaya et al., 2009). Why viruses from different genera have evolved proteins with similar structural and, apparently, functional roles, while only one member of the genus *Closterovirus* (CTV) has followed this evolutionary pathway, remains an intriguing conundrum.

## Conflict of Interest Statement

The authors declare that the research was conducted in the absence of any commercial or financial relationships that could be construed as a potential conflict of interest.
